# Fatigue Life Prediction of Machined Specimens with the Consideration of Surface Roughness

**DOI:** 10.3390/ma14185420

**Published:** 2021-09-19

**Authors:** Xiaochun Zhu, Zhurong Dong, Yachen Zhang, Zhengkun Cheng

**Affiliations:** School of Automotive and Transportation Engineering, Shenzhen Polytechnic, Shenzhen 518055, China; zhuxc@szpt.edu.cn (X.Z.); arondong@szpt.edu.cn (Z.D.); zhangyachen@szpt.edu.cn (Y.Z.)

**Keywords:** bending fatigue test, surface roughness, fatigue notch factor, fatigue life prediction

## Abstract

The fatigue strength and fatigue life of high-strength steels are greatly affected by their surface roughness. This study investigates the underlying mechanisms responsible for fatigue failure of the high-strength steel 42CrMo. Bending fatigue tests of stepped shafts with different levels of surface roughness were conducted to observe the fatigue live reduction affected by surface topography. Besides, the mechanical properties of 42CrMo and its strain–life relationship were established. Moreover, the analytical formulas to describe the stress concentration factor (SCF) and fatigue notch factor (FNF) induced by surface topography were introduced. To estimate the fatigue life of machined specimens with the consideration of surface roughness, the elastic portion of the total strain–life curve of the material was revised with the proposed analytical FNF imposed by surface topography. Comparisons between the estimated fatigue lives and experimentally obtained fatigue lives show that the effect of surface roughness on fatigue lives could be estimated effectively and conveniently by the proposed procedure.

## 1. Introduction

With high fatigue strength and good impact resistance, high-strength steel such as 42CrMo steel has been widely used as crankshaft materials. The fatigue strength evaluation and fatigue life prediction of crankshafts play a significant role in determining the reliability of engines and vehicles. It is widely recognized that the fatigue performance of high-strength steels is greatly affected by their surface roughness [1]. In general, the fatigue strength of engineering components increases with a decrease of surface roughness [2]. In engineering design and analysis, the effects of surface roughness are usually considered by using an empirical surface finish factor, which was defined for each type of machining process and commonly used to modify the fatigue limit of metals [1]. However, the empirical modification factors used in engineering fatigue design are mostly based on data published in the 1930s and 1940s, which are too conservative to estimate fatigue strength and can also result in increased cost [3].

Surface roughness introduces microscopic stress concentrations which can trigger crack initiation, especially for notch-sensitive materials and cases of high-cycle fatigue [4]. In terms of stress concentration induced by surface roughness, some empirical expressions based on standard surface roughness parameters were proposed, such as the Neuber rule [5] and the Arola–Ramulu model [6]. The finite element descriptions of surface topography to calculate the SCF were then put forward [7,8,9,10,11]. Recently, the theoretical formulas to obtain the SCF imposed by surface morphology based on Fourier representations were built as well [12,13,14,15]. Nevertheless, the SCF introduced by surface roughness is not enough to estimate the fatigue strength of rough specimens due to notch effects [16]. Therefore, the SCF was replaced by the FNF proposed by Neuber [1], Peterson [1], Siebel and Stieler [17] to evaluate the degradation of fatigue limits affected by surface roughness [3,7,9,18]. Apart from that, surface topography was simplified as multi-cracks by some researchers as well [3,4,19,20]. Taylor and Clancy considered that the short crack threshold model proposed by EI Haddad can be used to predict the fatigue limit of specimens with relatively low roughness levels, while the notch-based approach was suggested for those specimens with higher roughness levels [4]. Murakami and Endo [19] developed a formula to predict the fatigue limit of specimens with small defects and inclusions based on material hardness $H_{v}$ and the parameter $\sqrt{area}$, where the parameter $\sqrt{area}$ is related to the projection area of defects or inclusions, and they showed that the proposed formula was applicable to estimate the fatigue strength reduction induced by surface roughness [20].

To evaluate the effect of surface roughness on fatigue life of specimens, the slope of the elastic portion of the strain–life curve was revised by incorporating the FNF estimated by surface roughness parameters, and the estimated fatigue lives fall within the scatter bands of two compared with the observed fatigue test results [21]. Based on nonlinear finite element analysis (FEA) and the theory of critical distance (TCD), the SCF and stress gradient induced by surface roughness was calculated, the stress and strain over a critical distance were averaged and substituted into the Smith–Watson–Topper (SWT) model to estimate the fatigue lives of crack initiation of additively manufactured Ti6Al4V; the predicted fatigue cycles based on the proposed approach yield good accordance to the experimental data [22,23]. Besides, linear elastic fracture mechanics was applied by other researchers to analyze the fatigue life reduction influenced by surface roughness [10,24,25,26]. Suraratchai predicted the fatigue life of crack initiation and propagation for rough specimens based on the Basquin equation and Pairs law by incorporating the SCF calculated from the finite element model (FEM) of surface topography [10]. The stress intensity factor expressed with the equivalent defect size $\sqrt{area}$ induced by surface topography was calculated and combined with Pairs law to predict the fatigue lives of additively manufactured metals, and fatigue life predictions satisfied the experimental results well [25].

However, these proposed empirical equations based on surface roughness characterization parameters cannot be easily applied to describe the stress concentrations induced by surface morphology, because the real machined surface contains complex geometrical information which may not be suitable to be simplified as multi-notches or multi-cracks. Besides, the standard surface roughness parameters depend strongly on the resolution of the roughness-measuring instruments. While the FEM-based analysis of the measured surface morphology is a relatively time-consuming way to obtain the stress distribution, it also requires a new calculation for each different surface. By contrast, combined with the fatigue life prediction model, the analytical solutions to describe the SCF and FNF induced by surface topographies can provide an effective and convenient way to evaluate the fatigue lives reduction affected by surface roughness.

The organization of this paper is as follows. First, the bending fatigue tests to observe the effect of surface topographies on fatigue lives of specimens with different levels of surface roughness were carried out. The mechanical properties and strain–life curve of the 42CrMo steel were then investigated, the FEA of specimens under bending load was conducted as well. Based on the FEA results and the strain–life curve of the material, the fatigue life of the specimen without considering surface roughness effects was preliminarily estimated. Finally, the analytical solutions of the SCFs and FNFs induced by surface topography were introduced to estimate the fatigue limit reduction, the analytical FNFs were employed to revise the strain–life relationship to predict the fatigue lives of specimens with the consideration of surface roughness. Fatigue life prediction using the proposed method falls within scatter bands of two from experimentally obtained lives for specimens. The comparison between the estimated fatigue lives and experimentally obtained fatigue lives shows that the proposed procedure provided a powerful tool to evaluate the fatigue lives reduction affected by surface roughness.

## 2. Bending Fatigue Test

The initial motivation of this work aims to evaluate the effect of surface roughness on fatigue behaviors of the crankshaft. As several fracture incidents occurred due to poor machined surface conditions around the fillet of crankshafts [27,28], it is essential to have quantitative methods to account for surface roughness in fatigue life prediction of crankshafts. Reports show that bending fatigue fractures account for 80% of the failure cases for crankshafts [29], thus it is necessary to investigate the bending fatigue behaviors of crankshafts with the surface roughness effects. However, the manufacturing of crankshafts is time-consuming due to their complex geometry and complicated processes. Besides, the fabricating cost of crankshafts is too high. Here, the bending fatigue test of crankshafts was replaced as the bending fatigue test of stepped shafts, the test procedure is as follows.

**Figure 1:**
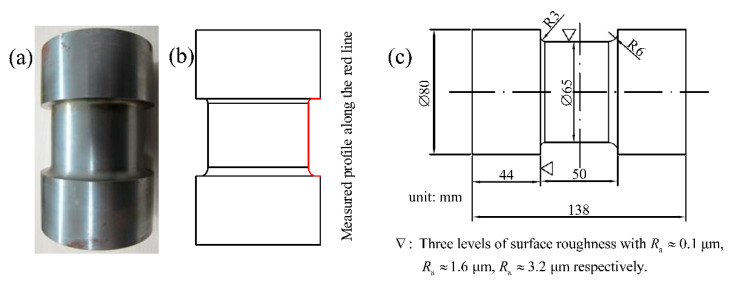
The fatigue test specimen. (**a**) the machined stepped shaft, (**b**) the schematic describing the profiled roughness and (**c**) the geometric dimensions of the stepped shaft.

Nine stepped shafts with three different levels of surface roughness on fillets were machined by turning. The Cincinnati HAWK TC150 CNC lathe was used for the turning, and the lathe cutting tool was made of KENNAMETAL WC-Tic-Tac (NbC)-Co carbide alloy. To guarantee the consistency of the dimensional precision for each workpiece, a coarse turning with the same cutting conditions was carried out for each specimen first. A final turning with different cutting parameters was then processed to obtain different levels of surface roughness on fillets for stepped shafts. Surface morphologies near the fillets of these stepped shafts were measured by using the TR300 stylus roughness measuring instrument. The roughness parameters, such as the average roughness, $R_{a}=\frac{1}{L}\int_{0}^{L}\left|Z\left(x\right)\right|dx$, the maximum peak-to-valley height, $R_{y}=\left|Z_{\max}-Z_{\min}\right|$ and the 10-point roughness, $R_{z}=\frac{1}{5}\sum_{i=1}^{5}{\left(Z_{i}\right)}_{\max}+\frac{1}{5}\sum_{j=1}^{5}{\left(Z_{i}\right)}_{\min}$, were used as characteristic parameters for the machined surface profiles, where *L* is the measured length, *Z* is the recorded profile; and *i* and j denote the highest peaks and lowest valleys, respectively [7,30]. The average roughness, $R_{a}$, of these three different levels of surface topographies are about 0.1 μm, 1.6 μm and 3.2 μm, respectively. To achieve these three different levels of surface roughness, the depth of cutting was set as 0.2 mm for all specimens. Other cutting conditions for these three levels of surface roughness were defined as follows: (a) the nose radius was 0.8 mm and the feed rate was 0.1 mm/r for specimens with $R_{a}\approx 0.1\;\mu m$; (b) the nose radius was 0.8 mm and the feed rate was 0.3 mm/r for specimens with $R_{a}\approx 1.6\;\mu m$; and (c) the nose radius was 0.4 mm and the feed rate was 0.3 mm/r for specimens with $R_{a}\approx 3.2\;\mu m$. Residual stress induced by the machining process for all specimens was relieved by annealing treatment. Figure 1 shows the machined stepped shaft, the schematic describing the profiled roughness and the geometric dimensions of the stepped shaft.

**Figure 2:**
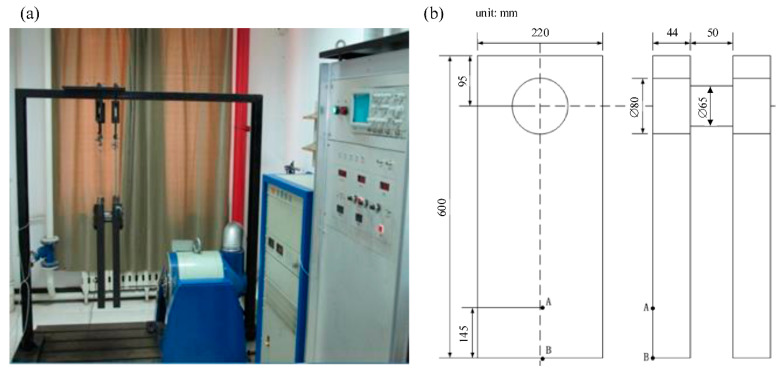
(**a**) The electric-resonant fatigue testing bench and (**b**) the dimensions of the resonant system.

The PDC-2 electric-resonant fatigue testing equipment was used to test the bending fatigue behavior of stepped shafts; the fatigue test bench and its dimensions are shown in Figure 2. The material of the stepped shafts is 42CrMo, the chemical compositions of 42CrMo were measured and are shown in Table 1, the material of the resonance plate is 45 steel.

The basic mechanical properties of 42CrMo and 45 steel are listed in Table 2 [31,32], where E and ν are the elasticity modulus and Poisson ratio, ρ is the density of the material, $\sigma_{s}$ and $\sigma_{b}$ are yield strength and ultimate strength of the material and ψ is the reduction of the area from a uniaxial tensile test.

**Figure 3:**
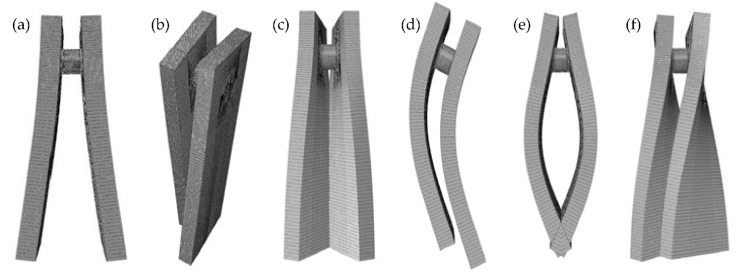
The modal shapes of the resonant system calculated by FEA. (**a**) 1st order, (**b**) 2nd order, (**c**) 3rd order, (**d**) 4th order, (**e**) 5th order, (**f**) 6th order.

The bending fatigue test system will be resonated when the excitation frequency equals the bending modal frequency of the resonant system, which can be simplified as a forced vibration system with two degrees of freedom. To determine the excitation frequency, the finite element modal calculation and frequency-sweeping test of the resonant system were performed. Figure 3 shows the modal shapes of the resonant system. The modal frequencies of the resonant system obtained by FEA and frequency-sweeping test are shown in Table 3. The relative error of the bending modal frequency of the resonant system obtained by two methods is less than 1%, which indicates that the bending modal frequency obtained by two methods is accurate enough. Thus, the force-frequency excited by the vibration generator was set as 112 Hz to ensure that the bending modal of the resonant system can be activated. The exciting force of the exciter was set as 15 kN and the corresponding bending moment was 5400 N·m. The exciting force was determined by the dynamic and static strain calibration method [33]. The number of load cycles is considered as the fatigue crack initiation life of the stepped shafts once the frequency of the resonant system decreases by 1% [34].

The bending fatigue test results of stepped shafts are shown in Table 4, the average roughness, $R_{a}$, peak-to-valley height roughness, $R_{y}$, and 10-point roughness, $R_{z}$, of each stepped shaft were measured and listed in Table 4 as well. The bending moment was 5400 N·m for all specimens. Experimental observation shows that the bending fatigue lives of stepped shafts with larger surface height parameters near the fillets are much shorter than those specimens with relatively smooth surface morphologies, which is consistent with most previous reports that the height parameters of surface topographies following machining are the most significant parameters to metal fatigue behaviors [3,4,10,18].

## 3. Fatigue Life Prediction of the Smooth Stepped Shaft

### 3.1. Fatigue Properties of 42CrMo

The strain-based approach is widely used to estimate the fatigue behavior of notched parts. As for the stepped shafts subjected to cyclic external loads, the mechanical behavior of the material at the root of the fillet is best considered in terms of strain. The total strain amplitude has been divided into elastic and plastic strain components from the steady-state hysteresis loops. The Ramberg–Osgood relationship shown in Equation (1) is used to describe the steady-state relationship between stress and strain.

(1) $$\varepsilon_{a}=\frac{\Delta \varepsilon_{e}}{2}+\frac{\Delta \varepsilon_{p}}{2}=\frac{\sigma_{a}}{E}+{\left(\frac{\sigma_{a}}{K^{\prime }}\right)}^{1/n^{\prime }},$$

where $\varepsilon_{a}$ is the strain amplitude, $\Delta \varepsilon_{e}/2$ and $\Delta \varepsilon_{p}/2$ are the elastic and plastic strain amplitudes, $\sigma_{a}$ is the stress amplitude, $K^{\prime }$ is the cyclic strength coefficient and $n^{\prime }$ is the cyclic strain hardening exponent [1].

At a given fatigue life, the total strain is the sum of the elastic and plastic strains, both the elastic and plastic curves can be approximated as straight lines. The strain–life data of smooth axial specimens is as follows:

(2) $$\varepsilon_{a}=\frac{\Delta \varepsilon_{e}}{2}+\frac{\Delta \varepsilon_{p}}{2}=\frac{\sigma_{f}^{\prime }}{E}{\left(2N_{f}\right)}^{b}+\varepsilon_{f}^{\prime }{\left(2N_{f}\right)}^{c},$$

where $\sigma_{f}^{\prime }$ and b are the fatigue strength coefficient and exponent and $\varepsilon_{f}^{\prime }$ and c are the fatigue ductility coefficient and exponent [1]. These parameters can be obtained from the following equations [31,35]:

(3) $$n^{\prime }=b/c,$$

(4) $$K^{\prime }=\sigma_{f}^{\prime }/{\left(\varepsilon_{f}^{\prime }\right)}^{n^{\prime }},$$

(5) $$b=-\frac{1}{6}\mathrm{lg}\left(\frac{2\sigma_{f}^{\prime }}{\sigma_{b}}\right),$$

(6) $$\sigma_{f}^{\prime }=1.19\sigma_{b}{\left(\sigma_{f}/\sigma_{b}\right)}^{0.893},$$

(7) $$\varepsilon_{f}^{\prime }=0.63\varepsilon_{f}{\left(1-81.8\left(\sigma_{b}/E\right){\left(\sigma_{f}/\sigma_{b}\right)}^{0.179}\right)}^{-1/3},$$

where $\sigma_{f}$ is the true fracture strength and $\varepsilon_{f}$ is the true fracture ductility; they can be obtained by the following equations:

(8) $$\sigma_{f}=\sigma_{b}+344.75,$$

(9) $$\varepsilon_{f}=\ln\left(\frac{1}{1-\psi }\right).$$

42CrMo steel is a cyclic softening material, so the fatigue ductility exponent is estimated as c=−0.609 [31,35]; other fatigue parameters used to predict the fatigue life of the material can be obtained by Equations (2)–(9), which are shown in Table 5.

**Figure 4:**
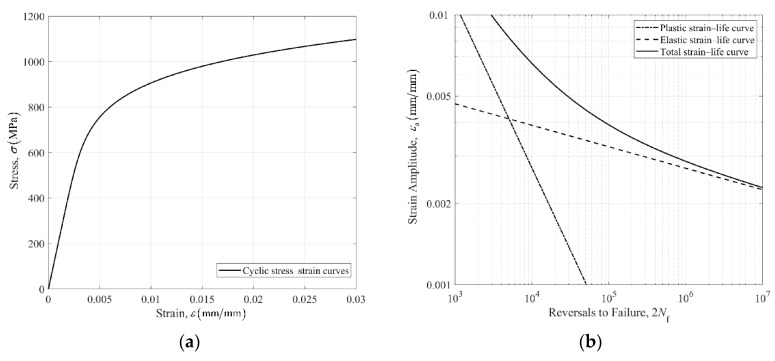
(**a**) Cyclic stress–strain curve of 42CrMo based on the Ramberg–Osgood relationship, (**b**) strain–life curve of 42CrMo based on Manson–Coffin equation.

The cyclic stress–strain curve of 42CrMo obtained by the Ramberg–Osgood relationship is shown in Figure 4a. The strain–life curves of 42CrMo obtained by the fatigue prediction parameters listed in Table 5 are shown in Figure 4b. The solid line in Figure 4b describes the total strain–life curve for the high strength steel 42CrMo, the dashed line and dash-dot line represent the elastic and plastic strain–life fitted curves, respectively.

### 3.2. FEA of the Stepped Shaft

**Figure 5:**
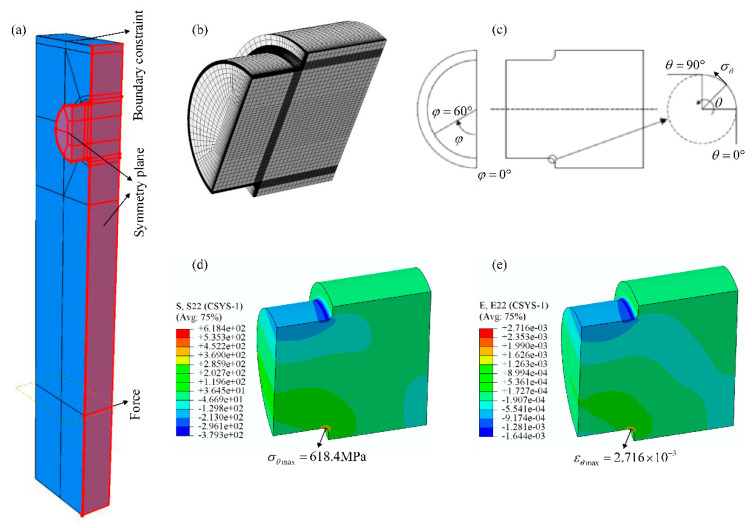
FEA to obtain the stress and strain distribution of the stepped shaft. (**a**) the 1/4 symmetry model of the resonant system, (**b**) the FEM of the 1/4 symmetry stepped shaft, (**c**) the schematic describing the nodes position near the fillet of stepped shaft, (**d**) the stress distribution of the stepped shaft, (**e**) the strain distribution of the stepped shaft.

To predict the fatigue life of stepped shafts under cyclic bending load, the stress and strain distribution near fillets of the specimens under bending load were investigated by FEA. Based on the symmetry of geometry and load boundary for the bending fatigue test system, the resonant system was simplified as a 1/4 symmetry model, shown in Figure 5a. The interface between the stepped shaft and the resonance plate was tied together. The FEM of the 1/4 symmetry stepped shaft was built, shown in Figure 5b, quadratic hexahedral elements were used for the FEM, mesh refinement was carried out near the fillet to satisfy the requirement of numerical convergence of stress distribution. Symmetry plane constraint and the top-side boundary constraint were imposed, and a half of the exciting force, 7.5 kN, was applied on the exciter site of the model shown in Figure 5a. The linear-elastic mechanics parameters of 45 steel shown in Table 2 and the steady-state stress–strain parameters of 42CrMo shown in Table 5 were employed to calculate the stress and strain distribution of the stepped shaft. Parameters φ and θ were used to describe the position of the nodes near the fillets of stepped shafts, which are shown in Figure 5c. φ represents the rotation angle around the axis of the stepped shaft and θ indicates the rotation angle of the circular arc around the fillet section of the stepped shaft; the stress component $\sigma_{\theta }$ along the θ direction is the bending principal stress.

The bending principal stress and strain of the stepped shaft under 5400 N·m bending moment are shown in Figure 5d,e. The maximum bending principal stress and strain of stepped shafts locate around the node position, $\varphi =0^{{}^{\circ}},\theta =60^{{}^{\circ}}$, which is consistent with the fatigue crack initiation sites of stepped shafts. The maximum bending principal stress $\sigma_{\theta \max}$ of the stepped shaft is 618.4 MPa, which is much less than the yield stress of 42CrMo. Therefore, the fatigue lives of the stepped shaft without considering the surface roughness effect should be located in the high-cycle fatigue regime.

The mean bending principal strain at the dangerous point around the fillet of the stepped shaft is zero during the bending fatigue test. By setting the maximum bending principal strain, $\varepsilon_{\theta \max}$, shown in Figure 5e as the strain amplitude and substituting it into Equation (2), the fatigue lives of the smooth stepped shafts under cyclic bending load can be predicted; the predicted fatigue life of the smooth stepped shaft is 1,662,300 reversals. Noting that the predicted fatigue life of the stepped shaft was not considered to the effect of surface roughness.

## 4. Fatigue Life Estimations Based on Surface Roughness

### 4.1. Fatigue Notch Factor of Rough Specimens

**Figure 6:**
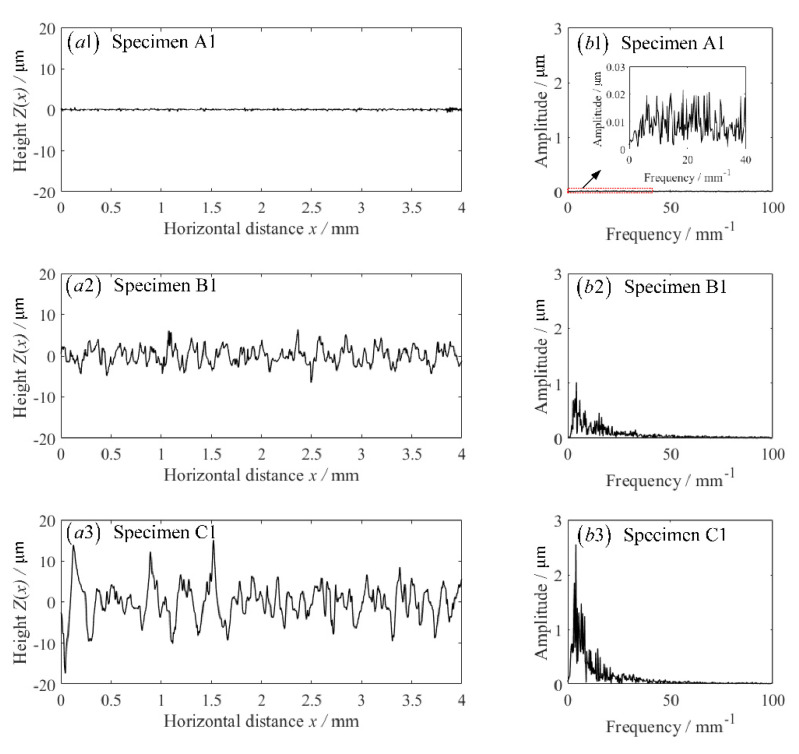
Machined surface topographies and their amplitude-frequency analysis. (**a1**,**a2**,**a3**) the machined surface topographies measured from specimens A1, A2 and A3; (**b1**,**b2**,**b3**) the amplitude-frequency relationships for the machined surface topographies in (**a1**,**a2**,**a3**).

Surface topographies nearby the fillets along the axial direction of these stepped shafts were measured by TR300 stylus roughness measuring instrument, the raw data of the measured surface profiles were extracted and analyzed by Fourier transform. Figure 6 shows the machined surface topographies near the fillets along the axis direction of three stepped shafts and their amplitude-frequency analysis results. Results show that surface topographies with higher $R_{a}$ are predominant due to their low frequency components.

The machined surface topography can be assumed to be a stationary stochastic process, and it can be modelled based on superposing a series of cosine components through Fourier transform [36,37]. The corresponding Fourier series can be formulated as below:

(10) $$Z(x)=-\sum_{i=1}^{n}A_{i}\cos\left(2\pi x/\lambda_{i}+\varphi_{i}\right),$$

where $A_{i}$ is the amplitude of the *i*-th wave, $\lambda_{i}$ is the wavelength of the *i*-th wave and $\varphi_{i}$ is the phase of the *i*-th wave. 

Surface morphologies can be considered as multi-notches, which can introduce stress concentrations and affect the fatigue behaviors of specimens. The SCFs induced by surface topography are as follows [12]:

(11) $$K_{t}(x)=1+4\pi \sum_{i=1}^{n}\frac{A_{i}}{\lambda_{i}}\cos\left(\frac{2\pi x}{\lambda_{i}}+\varphi_{i}\right).$$

Real surface topography can be thought of as a series of notches of varying sizes and shapes, so the TCD can be used to estimate the fatigue limit of rough specimens. The reference stress used to predict the FNF of notched components is not the stress at the root of the notch but a point stress at a given distance ahead of the notch, corresponding to the point method (PM) of TCD [38]. According to the PM of TCD, notched parts are in their fatigue limit condition when the effective stress, $\Delta \sigma_{\mathrm{eff}}$, which depends on the maximum principal stress at a distance from the notch tip of $a_{0}/2$, equals the material fatigue limit, $\Delta \sigma_{0}$ [38]. The FNFs of rough specimens were derived by combing the analytical solutions of the stress distribution induced by surface topographies and the PM of TCD, which are as follows [13]:

(12) $$K_{f}=1+4\pi \sum_{i=1}^{n}\frac{A_{i}}{\lambda_{i}}\left(1-\frac{\pi a_{0}}{2\lambda_{i}}\right)e^{-\pi a_{0}/\lambda_{i}}\cos\left(\frac{2\pi x_{*}}{\lambda_{i}}+\varphi_{i}\right),$$

where $x_{*}$ is the notch’s bottom position of surface topography, $a_{0}$ is the material characteristic length, which is defined by the fatigue crack growth threshold, $\Delta K_{\mathrm{th}}$, and the material fatigue limit, $\Delta \sigma_{0}$, $a_{0}={\left(\Delta K_{\mathrm{th}}/\Delta \sigma_{0}\right)}^{2}/\pi$; $a_{0}$ is a material parameter which is only dependent on the types of materials and load ratios [39].

The material characteristic length $a_{0}$ varies considerably for different materials, commonly encountered values range from microns to millimeters. High-strength steels, including 42CrMo, were recognized to have an $a_{0}$ value of about 10 μm [40,41]. According to Equation (12), the high frequency cut-off of machined surface topography can be defined as follows [13]:

(13) $$f_{\mathrm{cut}-\mathrm{off}}=\frac{2}{\pi a_{0}},$$

where the surface frequency components, $f_{i}=1/\lambda_{i}$, surface wave components with frequencies higher than $f_{\mathrm{cut}-\mathrm{off}}$ can be removed, because they make no contributions to the stress raiser at the reference point. Besides, to model the effect of surface roughness on the fatigue behavior of metals, more high frequency components have to be considered to reconstruct the machined surface topographies for high strength steels compared to some materials with less sensitivity to surface roughness.

**Figure 7:**
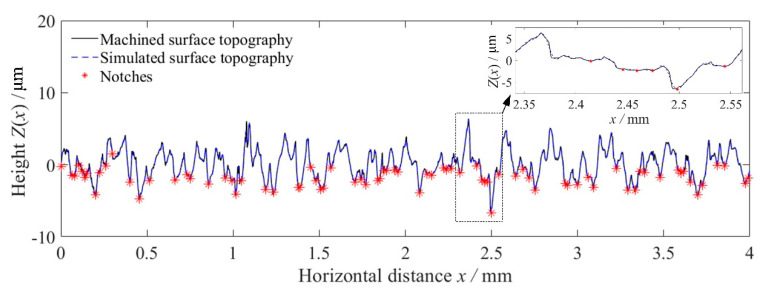
Machined surface topography and simulated surface topography for specimen B1.

**Figure 8:**
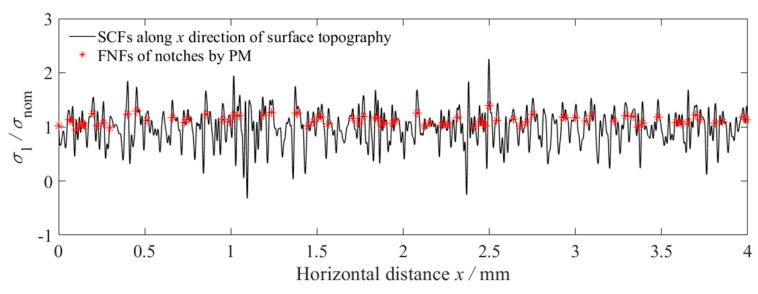
The SCFs and FNFs induced by surface topography for specimen B1.

Based on Equations (10)–(13), the high frequency cut-off is defined as $f_{\mathrm{cut}-\mathrm{off}}=63.7\;{\mathrm{mm}}^{-1},$ the machined surface topographies around the fillets of these stepped shafts were reconstructed by Fourier series, and the SCFs and FNFs of these reconstructed surface topographies were calculated. Figure 7 shows the machined surface topography and the reconstructed surface topography for specimen B1. Figure 8 shows the SCFs induced by the reconstructed surface topography and the FNFs by PM for each notch extracted from the reconstructed surface topography for specimen B1. It was found that the maximum SCF reaches 2.25 while the maximum FNF is only about 1.39.

Surface topographies act as multi-notches to introduce stress concentration and affect the fatigue performance of specimens. By analogy with the characterization of surface roughness, the maximum SCF, $K_{\mathrm{tmax}}$, the effective SCF, ${\bar{K}}_{t}$ by averaging the top 10 values, the maximum FNF, $K_{\mathrm{fmax}}$, and the effective FNF, ${\bar{K}}_{f}$, by averaging the top 10 values are defined as the characteristic parameters to represent the fatigue behavior of rough specimens, where ${\bar{K}}_{t}=\frac{1}{10}\sum_{i=1}^{10}{\left(K_{ti}\right)}_{\max}$, ${\bar{K}}_{f}=\frac{1}{10}\sum_{i=1}^{10}{\left(K_{fi}\right)}_{\max}$. Figure 8 shows that the maximum FNF, $K_{\mathrm{fmax}}=1.39$, and the effective FNF, ${\bar{K}}_{f}=1.27$. 

According to Equations (10)–(13), the SCFs and FNFs imposed by surface topographies of each specimen are calculated and shown in Table 6. It is clear that the SCFs are much higher than the FNFs of each specimen. Besides, the maximum FNF, $K_{\mathrm{fmax}}$, and the effective FNF, ${\bar{K}}_{f}$, increase gradually with the increase of the average roughness, $R_{a}$. To quantitatively evaluate the effect of surface topography on the fatigue strength and fatigue life of rough specimens, the maximum FNF, $K_{\mathrm{fmax}}$, and the effective FNF, ${\bar{K}}_{f}$, for the same degree of surface roughness were averaged, which are shown in Table 6.

### 4.2. Fatigue Life Prediction of Rough Specimens

It is well established that the surface roughness has little influence on the fatigue behaviors in the low-cycle fatigue region, but affects the high-cycle fatigue regime more, where elastic strain is dominant. Therefore, only the elastic portion of the strain–life curve is modified to account for the effect of surface topography [1]. Based on the 10^7^ run-out reversals considered in this study, the fatigue limit of rough specimens is estimated with the method of dividing the fatigue limit of polished specimens by the FNF induced by surface topographies. Here, the fatigue limit of 42CrMo is predicted by subtituting 10^7^ reversals into Equation (2). 

Alternatively, the fatigue life of the rough specimen is predicted by adjusting the slope of the strain–life curve to capture the effect of surface roughness for parts that are not polished. With the consideration of surface topography, the total strain–life equation is revised as follows [21]:

(14) $$\varepsilon_{a}=\frac{\Delta \varepsilon_{e}}{2}+\frac{\Delta \varepsilon_{p}}{2}=\frac{\sigma_{f}^{\prime }}{E}{\left(2N_{f}\right)}^{b-\frac{1}{7}\log\left(K_{f}\right)}+\varepsilon_{f}^{\prime }{\left(2N_{f}\right)}^{c},$$

where $\sigma_{f}^{\prime }$, b, $\varepsilon_{f}^{\prime }$ and c are obtained from Table 5. The only information needed in this approach are the FNFs calculated by surface topographies from rough specimens.

**Figure 9:**
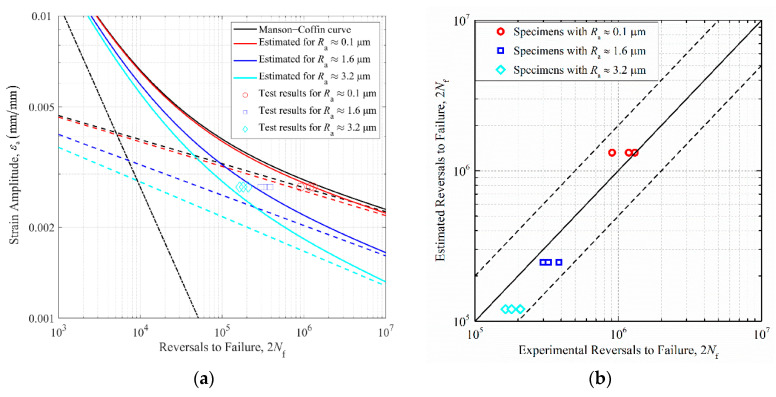
(**a**) Strain–life curve for 42CrMo steel and estimated curve for rough specimens based on correcting the elastic strain behavior using the average $K_{\mathrm{fmax}}$ induced by surface topographies. (**b**) Estimated fatigue lives compared to experimentally obtained fatigue lives with the scatter bands of two.

To quantitatively estimate the effect of surface topography on the fatigue life behaviors of stepped shafts, according to Equation (14), the elastic portion of the total strain–life curve of 42CrMo was solely adjusted based on the average $K_{\mathrm{fmax}}$ and the average ${\bar{K}}_{f}$ in Table 6. Figure 9a shows the strain–life curve for 42CrMo steel based on the prediction parameters in Table 5, and the estimated strain–life curve for rough stepped shafts based on adjusting the slop of elastic strain–life line using the average $K_{\mathrm{fmax}}$ listed in Table 6. The black dashed line and dash-dot line represent the elastic and plastic strain–life lines of 42CrMo steel. The red dashed line and solid line represent the estimated elastic and total strain–life curves of specimens with $R_{a}\approx 0.1\;\mu m$; the blue dashed line and solid line represent the estimated elastic and total strain–life curves of specimens with $R_{a}\approx 1.6\;\mu m$; the cyan dashed line and solid line represent the estimated elastic and total strain–life curves of specimens with $R_{a}\approx 3.2\;\mu m$. The predicted versus the experimentally observed fatigue lives of rough stepped shafts with scatter bands of two are shown in Figure 9b. All data points fall within the scatter band of two, demonstrating the effectiveness of estimating the fatigue life of rough specimens based on the procedure proposed above. 

**Figure 10:**
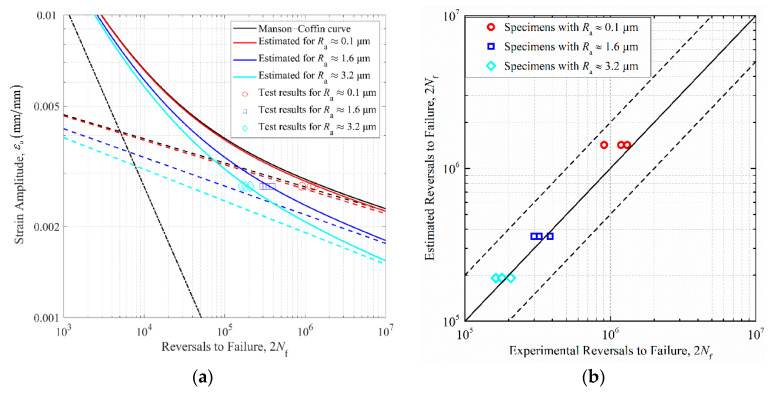
(**a**) Strain–life curve for 42CrMo steel and estimated curve for rough specimens based on correcting the elastic strain behavior using the average ${\bar{K}}_{f}$ induced by surface topographies. (**b**) Estimated fatigue lives compared to experimentally obtained fatigue lives with the scatter bands of two.

Figure 10a describes the strain–life curve for 42CrMo steel and the estimated strain–life curve for rough specimens by revising the fatigue strength exponent using the average ${\bar{K}}_{f}$ listed in Table 6. The estimated versus the fatigue test results of rough stepped shafts within a scatter band of two is shown in Figure 10b. All estimated fatigue lives again fall within the scatter bands of two compared with the observed fatigue test results. The proposed two methods demonstrate that surface topographies with $R_{a}\approx 0.1\;\mu m$ make little contribution to the fatigue behavior of stepped shafts. In terms of the fatigue life prediction of rougher stepped shafts, fatigue strength estimation using the effective FNF shows higher prediction accuracy than another approach by employing the maximum FNF. The comparison shows that the effective FNF is more likely to determine the fatigue performance of rough specimens than the maximum FNF induced by surface morphologies.

## 5. Conclusions

The main purpose of this research was to assess the effect of surface topography on the fatigue behaviors of high-strength steel. To achieve the objective of this work, the bending fatigue test of rough stepped shafts was carried out first. The fatigue properties of 42CrMo were then estimated, and the stress and strain distribution near the fillet of the stepped shaft under bending load were investigated by FEA. Lastly, based on the analytical formulas of the FNF induced by surface topography, the fatigue life of rough specimens was evaluated with a revised strain–life prediction model. Besides, the prediction accuracy of the revised strain–life model was validated by the bending fatigue test results. Some impactful conclusions were drawn as below:

(1) The fatigue lives decrease gradually with the increase of the height parameters of surface topographies according to the fatigue test. Besides, the FNF induced by surface roughness also conforms to this rule from the theoretical perspective. Theoretical and experimental results all point out that the height parameters of surface irregularities following machining are the most significant roughness parameters to characterize the fatigue performance of the material.
(2) The effect of surface roughness on the metal’s fatigue behavior can be adequately estimated using the analytical FNF based on the PM of TCD. The proposed FNF induced by surface topography is represented by the Fourier series and incorporated into the characteristic length $a_{0}$. According to the proposed procedure, more high frequency components of surface morphology should be taken into account to model the machined surface topography for high-strength steels, compared to low notch sensitive materials.
(3) A classical approach utilizing the FNF induced by surface topography to revise the elastic portion of the total strain–life curve can adequately capture the surface roughness effects. Fatigue life prediction using this method falls within scatter bands of two from experimentally obtained lives for specimens. Besides, the effective FNF is more suitable to characterize the fatigue performance of rough specimens than the maximum FNF induced by surface morphologies.

## Figures and Tables

**Table 1:** Chemical compositions of 42CrMo steel (wt.%).

C	Si	Mn	S	P	Cr	Ni	Cu	Mo
0.40	0.31	0.67	0.02	0.01	1.05	0.02	0.01	0.20

**Table 2:** Mechanical properties of 42CrMo and 45 steel.

	E(GPa)	ν	$\rho \left(\mathrm{kg}/m^{3}\right)$	$\sigma_{s}\left(\mathrm{MPa}\right)$	$\sigma_{b}\left(\mathrm{MPa}\right)$	ψ(%)
42CrMo	211	0.28	7850	930	1134	62
45 steel	206	0.3	7850	385	655	52

**Table 3:** Modal results of the resonant system.

	Order	1	2	3	4	5	6
Modal calculation	Frequency/Hz	112.74	181.50	488.43	504.02	728.88	853.77
Modal test		112.16	179.75	484.68	496.37	709.26	824.15

**Table 4:** Bending fatigue test results of stepped shafts.

Specimen ID	$R_{a}\left(\mu m\right)$	$R_{y}\left(\mu m\right)$	$R_{z}\left(\mu m\right)$	Bending Load (N·m)	$2N_{f}\left(\mathrm{Reversals}\right)$
A1	0.10	1.44	0.78	5400	906,400
A2	0.11	1.36	0.69	5400	1,306,200
A3	0.09	0.82	0.47	5400	1,185,400
B1	1.63	12.95	8.70	5400	325,200
B2	1.61	10.81	7.20	5400	386,300
B3	1.60	13.48	6.98	5400	298,800
C1	3.29	32.46	15.83	5400	207,400
C2	3.25	25.01	12.78	5400	163,400
C3	3.21	24.12	10.25	5400	180,300

**Table 5:** Fatigue life prediction parameters of 42CrMo steel.

$\sigma_{f}\left(\mathrm{MPa}\right)$	$\varepsilon_{f}$	$\sigma_{f}^{\prime }\left(\mathrm{MPa}\right)$	$\varepsilon_{f}^{\prime }$	b	c	$n^{\prime }$	$K^{\prime }\left(\mathrm{MPa}\right)$
1478.75	0.9676	1710.4	0.7385	−0.0795	−0.609	0.1305	1779.4

**Table 6:** The SCFs and FNFs induced by surface topographies of specimens.

Specimen ID	$R_{a}\left(\mu m\right)$	$K_{\mathrm{tmax}}$	${\bar{K}}_{t}$	$K_{\mathrm{fmax}}$	${\bar{K}}_{f}$	$\mathrm{Average}\;K_{\mathrm{fmax}}$	$\mathrm{Average}\;{\bar{K}}_{f}$
A1	0.10	1.21	1.20	1.03	1.02	1.03	1.02
A2	0.11	1.28	1.24	1.04	1.03		
A3	0.09	1.21	1.19	1.03	1.02		
B1	1.63	2.25	2.19	1.39	1.27	1.40	1.28
B2	1.61	2.97	2.85	1.45	1.31		
B3	1.60	2.63	2.51	1.37	1.25		
C1	3.29	3.31	3.23	2.01	1.54	1.76	1.50
C2	3.25	2.84	2.79	1.71	1.50		
C3	3.21	2.61	2.59	1.57	1.45		

## Data Availability

The data presented in this study are available on request from the corresponding author.
